# Left ventricular hypertrabeculation is a novel predictor of life-threatening arrhythmic events in long QT syndrome patients

**DOI:** 10.1186/s13023-025-04172-7

**Published:** 2025-12-22

**Authors:** Jing Yang, Kun Li, Fang Liu, Lingyun Kong, Yuanwei Liu, Fei She, Rong He, Tingting Lv, Fulan Liu, Ping Zhang

**Affiliations:** 1 Department of Cardiology, Beijing Tsinghua Changgung Hospital, School of Clinical Medicine, Tsinghua Medicine, Tsinghua University, Beijing, 102218 China; 2 Department of Cardiology, Peking University People’s Hospital, Peking University, Beijing, 100014 China

**Keywords:** Long QT syndrome, Left ventricular hypertrabeculation, Clinical presentations, Genetic characteristics, Life-threatening arrhythmic events

## Abstract

**Background:**

Long QT syndrome (LQTS) is a malignant cardiac channelopathy for patients with traditionally normal heart structures. Sporadic cases reported left ventricular hypertrabeculation (LVHT) was a deadly double hit in LQTS patients. Here we summarize the prevalence of LVHT within LQTS subjects and analyse the correlation of LVHT with life-threatening arrhythmic events (LAEs).

**Methods:**

The study cohort consisted of 170 genotype-confirmed LQTS patients, which were classified to two groups, including non-left ventricular hypertrabeculation LQTS patients(LQTS-N-LVHT group) and LQTS-LVHT co-phenotype individuals (LQTS-LVHT group) according to the diagnosis criteria of LVHT. The primary endpoint was LAEs, defined as arrhythmogenic syncope, sustained ventricular tachycardia (VT), appropriate ICD shocks, sudden cardiac death.

**Results:**

Eight LQTS patients (8/170, 4.7%) in the cohort met diagnostic criteria of LVHT. No statistical difference in symptomatology, Schwartz score, QTc interval in electrocardiograms, and proportion of PVCs or torsade de points (Tdp) in ambulatory Holter monitoring was revealed between two groups. By contrast, LQTS-LVHT subjects had a higher ratio of documented VF in ambulatory Holter monitoring compared with LQTS-N-LVHT patients (LQTS-LVHT 2/8, 25.0% vs. LQTS-N-LVHT 3/111, 2.7%, *p* = 0.036). In total, 59 patients (59/170, 34.7%) developed LAEs during follow-up duration of 52.8 ± 32.3 months. Significant difference was found between two groups by the log-rank test (*p* < 0.001).A univariate Cox regression analysis was performed demonstrating that LVHT was a significant predictor of LAEs[HR: 4.02 (1.71–9.45), *P* = 0.001].A multivariate Cox regression analysis indicated that LVHT remained a predictor of LAEs [hazard ratio: 3.02 (1.24–7.36), *p* = 0.015].

**Conclusion:**

LVHT is a novel predictor of life-threatening arrhythmic events in LQTS patients. Effective prevention treatment should be emphasized to prevent life-threatening cardiac events in LQTS-LVHT patients.

**Supplementary Information:**

The online version contains supplementary material available at 10.1186/s13023-025-04172-7.

## Introduction

Long QT syndrome (LQTS) is a potentially fatal cardiac channelopathy characterized by QT interval prolongation and an increased risk of syncope or sudden cardiac death (SCD) in patients with traditionally normal heart structures [1]. Left ventricular hypertrabeculation (LVHT) is distinguished by excessive LV trabeculations and an endocardial noncompacted layer that is > 2 folds thicker than the epicardial compacted layer of the myocardium (NC/C > 2), which is thought to develop arrhythmia, heart failure, and embolism [2]. Recently, a few reported cases identified that LVHT can be co-phenotype with LQTS patients experiencing malignant events including recurrent syncope and SCD [3–8]. Herein, we summarize the prevalence of LVHT within a large cohort of LQTS subjects and compare the clinical, electrocardiographic and prognostic characteristics beween isolated LQTS and LQTS with LVHT patients.Then the correlation and clinical significance of LVHT with life-threatening arrhythmic events(LAEs) is analysed.

## Methods

### Definitions

The Heart Rhythm Society (HRS) expert consensus statement on the diagnosis and treatment of patients with inherited primary arrhythmia syndromes was applied to the diagnosis of LQTS [9]. There is no universal standard used in LVHT diagnosis. The conventional diagnostic criteria for echocardiography and cardiac magnetic resonance (CMR) rely primarily on the non-compacted layer to compact layer thickness ratio, color doppler echocardiogram evidence of inter-trabecular recesses filled from the LV cavity, and segmental localization of hypertrabeculation indicative of noncompaction. Our study’s diagnosis of LVHT was based on the echocardiography criteria put forward by Jenni et al. [10], and CMR criteria proposed by Petersen SE et al. [11](Supplementary Table 1).

### Study population

The study cohort consisted of 170 genotype-confirmed LQTS patients who were admitted to the cardiology department of Beijing Tsinghua Changgung Hospital and Peking University People’s Hospital during the year 2006 to 2024. Patients were classified to two groups, including LQTS isolated (non-left ventricular hypertrabeculation LQTS patients, LQTS-N-LVHT) group and LQTS-LVHT co-phenotype group, according to the the diagnosis criteria of LVHT by echocardiography or/and CMR examinations in the initial stage.

The study protocol was approved by the Ethics Committee of Beijing Tsinghua Changgung Hospital. The patient or the patient’s legal guardian submitted written informed consent following the Helsinki’s ethical guidelines.

### Clinical and electrocardiographic assessment

Retrospective review of the clinical record, a series of electrocardiograms (ECGs) (12-lead resting ECG, 24-hour ambulatory ECG) (GE Healthcare, Fairfield, America), medication treatment, and device implantation were performed. Patients were defined as symptomatic if they had a history of cardiac syncope, documented ventricular tachycardia, or a resuscitated cardiac arrest. Appropriate shocks recorded from implanted cardioverter-defibrillators (ICDs) were defined by qualified electrophysiological physicians.

All ECGs were analyzed by two experienced readers (Dr. Jing Yang and Ping Zhang) independently. The QT interval was measured from the start of the QRS complex to the end of the T wave in individual leads, which showed the longest QT should be used(usually V2 or V3) [12].The QTc (QT interval corrected for heart rate) was calculated by Bazett formulae[QTc= (HR/60)^1/2^ =QT (RR) ^1/2^]. The echocardiography and cardiac magnetic resonance (CMR) examinations were collected and diagnosed by two skilled professionals (Dr. Lingyun Kong and Fang Liu).

### Genetic assessment

Patients enrolled in this study were genetically confirmed with pathogenic mutations of reported 15 LQTS pathogenic genes (*KCNQ1*,* KCNH2*,* SCN5A*,* ANK2*,* KCNJ2*,* KCNE1*,* KCNE2*,* CACNA1C*,* CAV3*,* SCN4B*,* SNTA1*,* KCNJ5*,* CALM1*,* CALM2*,* CALM3*), and LVHT susceptibility genes (*LDB3*,* ACTC1*,* MYH7*,* TNNT2*,* TPM1*,* MYBPC3*, and *TAZ*) by Sanger sequencing. Family members of probands with disease-causing mutations completed family-specific genetic testing only. The gene variants were classified in likely pathogenic/pathogenic following the current American College of Medical Genetics and Genomics (ACMG) guidelines [13].

### Follow-up and outcomes

The primary endpoint was life-threatening arrhythmic events (LAEs), including arrhythmogenic syncope, sustained polymorphic ventricular tachycardia (VT), appropriate ICD shocks, aborted sudden cardiac death or sudden cardiac death. Syncope events were classified into probable arrhythmia-related (PAR) and unlikely arrhythmia-related syncope by two experienced cardiovascular physicians. The follow-up time was measured from the date of first definitely LQTS diagnosis or electrocardiographic confirmation of QTc interval prolongation to the first LAEs.

### Statistics

Baseline variables are shown as number (percentage) or mean ± standard deviation(SD). Comparison of categorical variables was performed by Fisher exact test. Independent-samples Student’s t-test was used to compare means. The Kaplan–Meier method was applied to estimate the cumulative probability for the occurrence of an outcome. To test the hypothesis of linearity of the relationship between the predictor (LVHT) and the outcome LAEs, univariable Cox regression models were built. A multivariate model that simultaneously controlled for age, sex, first symptom, Schwartz score, QTc interval in 12-lead ECGs,β-blocker and mexiletine/flecainide treatment. Statistics were considered significant at two-sided *p* < 0.05.

## Results

Among 170 genotype-confirmed LQTS patients (108 females, 63.5%) in our study, eight (8/170, 4.7%) LQTS patients met diagnostic criteria of LVHT. Clinical, electrocardiographic, therapeutic and prognostic characteristics of patients with isolated LQTS (*n* = 162) and LQTS-LVHT (*n* = 8) are summarized in Table 1.

**Table 1:** Clinical characteristics of LQTS, LQTS-N-LVHT and LQTS-LVHT cases

Demographic	LQTS (*n* = 170)	LQTS-N-LVHT(*n* = 162)	LQTS-LVHT(*n* = 8)	*p* value^a^
General/symptoms				
Female, n (%)	108/170(63.5)	102/162(63.0)	6/8(75.0)	0.712
Symptomatic^b^, n (%)	84/170(49.4)	79/162(48.8)	5/8(62.5)	0.493
SCA, n (%)	9/170(5.3)	8/162(4.9)	1/8(12.5)	0.359
Age of onset, year	18.9 ± 15.1	19.5 ± 15.3	10.1 ± 8.7	0.103
Age of diagnosis, year	27.9 ± 17.5	28.6 ± 17.4	14.4 ± 13.5	0.110
SCD family history, n(%)	45/170(26.5)	43/162(26.5)	2/8(25.0%)	1.0
LQTS family history, n(%)	75/170(44.1)	71/162(43.8)	4/8(50.0%)	0.733
Schwartz Score	4.7 ± 1.9	4.7 ± 1.9	5.6 ± 1.8	0.934
ECG				
QTc, ms	518.1 ± 64.1	517.4 ± 63.8	529.9 ± 72.7	0.978
Ambulatory Holter Monitoring^c^				
PVCs, n(%)	44/119(37.0)	41/111(36.9)	3/8(37.5)	1.0
Tdp, n (%)	21/119(17.6)	18/111(16.2)	3/8(37.5)	0.147
VF, n (%)	5/119(4.2)	3/111(2.7)	2/8(25.0)	0.036*
Therapy				
β-Blocker, n (%)	109/170(64.1)	102/162(63.0)	7/8(87.5)	0.261
Mexiletine//flecainide, n (%)	24/170(14.1)	21/162(13.0)	3/8(37.5)	0.086
ICD placement, n (%)	31/170(18.2)	28/162(17.3)	3/8(37.5)	0.161
Left cardiac sympathetic denervation, n (%)	11/170(6.5)	11/162(6.8)	0/8(0)	N/A
Follow-up				
LAEs^d^, n(%)	59/170(34.7)	53/162(32.7)	6/8(75.0)	0.022*

Abbreviations: ECG, electrocardiogram; ICD, implantable cardioverter-defibrillator; LAE, life-threatening arrhythmic event; LQTS, long qt syndrome; LQTS-N-LVHT, non-left ventricular hypertrabeculation in long qt syndrome patients; LQTS-LVHT, left ventricular hypertrabeculation in long qt syndrome patients; PVCs, premature ventricular contractions; QTc, heart rate-corrected QT interval; SCA, sudden cardiac arrest; SCD, sudden cardiac death; SNDD, standard diviation of NN intervals; Tdp, torsade de points; TWA, T-wave alternans; VF, ventricular fibrillation;

^a^ Statistical analysis compared LQTS-N-LVHT and LQTS-LVHT patients.

^b^ Statistical analysis compared LQTS-N-LVHT and LQTS-LVHT patients.

^c^ Ambulatory Holter monitoring data was obtained in 119/170 LQTS patients.

^d^ life-threatening arrhythmic event (LAE), including arrhythmogenic syncope, sustained polymorphic ventricular tachycardia(VT), appropriate ICD shocks, aborted sudden cardiac death or sudden cardiac death

### Clinical characteristics

No statistical difference in symptomatology, onset age, diagnosis age, SCD or LQTS family history and Schwartz Score was revealed between two groups. In LQTS-LVHT group, syncope or a resuscitated cardiac arrest after stress and/or exercise inducement was the first manifestation at the onset age of 10.1 ± 8.7 years. It should be mentioned that the frequency of syncope increased significantly after menophania in the female patient. The age of LQTS-LVHT diagnosis was 14.4 ± 13.5 years mainly because of misdiagnosis or ignorance. A LQTS-LVHT patient (case 2) was misdiagnosed with epilepsy for years because of convulsion of the limbs and eyes on the turn during syncope and was recommended to receive lobotomy surgery.

### Electrocardiographic characteristics

**Fig. 1:**
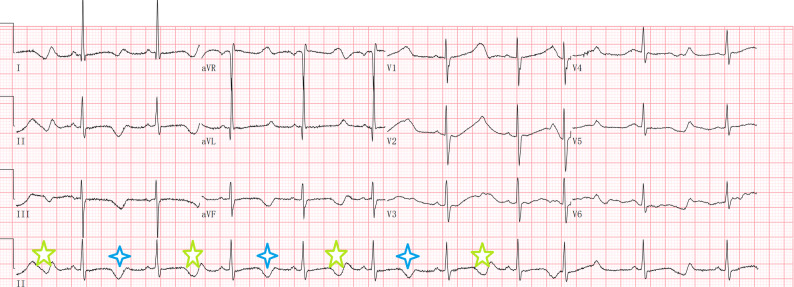
ECG of a LVHT-LQTS co-phenotype patient (case 5). PR segment interval and QRS wave duration were normal, QTc prolongated to 716 ms with T-wave alternans

**Fig. 2:**
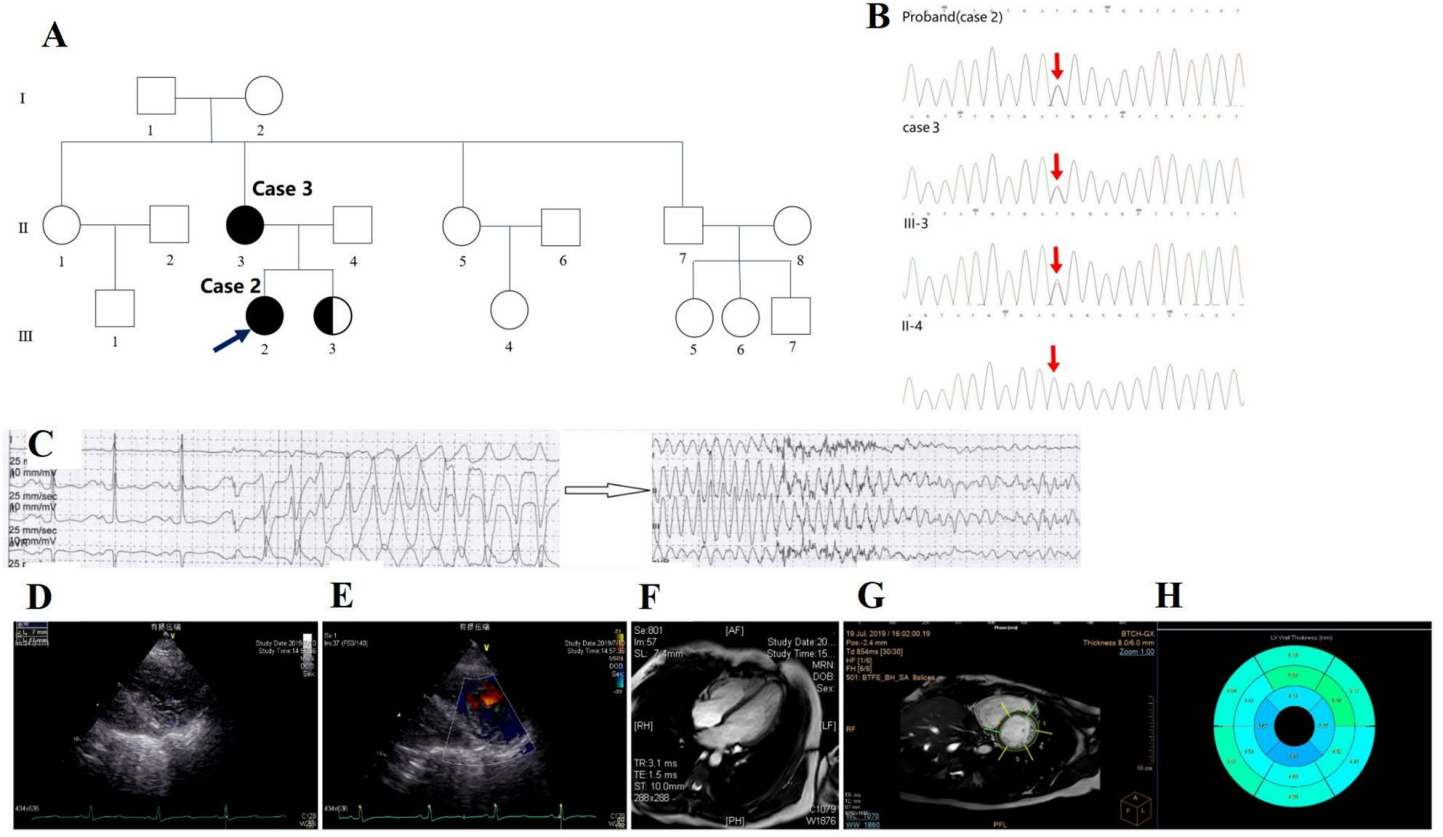
The images of LVHT co-phenotype(Case2). **(A.B)**Pedigree of Case 2,3(LQTS-LVHT) and genetic sequencing electropherogram.The sibling of the proband(III-3) was diagnosed with LQTS(QTc = 614ms) only carrying the same mutation *KCNH2* p.T273M.**(C)**24-hour-Holter of Case4 recorded Tdp deteriorating into ventricular fibrillation(VF). **(D.E)**Echocardiogram. **(D)**The thickness ratio of the non-compacted layer to the compacted layer on the short-axis image of the echocardiography satisfied the Stollberger et al. criteria1 at end-diastolic. **(E)** Intertrabecular recesses in the proband’s colour Doppler echocardiography that were filled with blood from the left ventricular cavity. **(F.G.H)** Cardiac magnetic resonance imaging (CMR) in case 2. **(F)** With a ratio of non-compacted to compacted myocardium of 3.4/1, the 4-chamber images on CMR demonstrated typical signs of left ventricular hypertrabeculation and met the Petersen et al. criteria. **(G.H)** Using a short-axis image of a two-chamber view and the standard AHA model, the myocardium was segmented into 17 sections

Analysis of QTc interval in electrocardiograms revealed no significant difference between two groups. In eight LQTS-LVHT individuals, QTc interval was prolonged to 529.9 ± 72.7 ms and T-wave alternans were present in Case 5 (Fig. 1). In the 170 genotype-confirmed LQTS patients, 119 (119/170, 70%) patients underwent 24-hour ambulatory monitoring. Of these, analysis of the proportion of PVCs and torsade de points (Tdp) indicated that LQTS-N-LVHT virtually indistinguishable from LQTS-LVHT patients. By contrast, LQTS-LVHT subjects had a higher ratio of documented VF in ambulatory Holter monitoring compared with LQTS-N-LVHT patients (LQTS-LVHT 2/8 25.0% vs. LQTS-N-LVHT 3/111 2.7%, *p* = 0.036). Tdp deteriorated into ventricular fibrillation (VF) for 5.5 sec in a LQTS-LVHT patient (case 2, Fig. 2C).

### Echocardiographic and CMR characteristics

The echocardiography examination in eight LQTS-LVHT patients revealed a median ejection fraction (EF) of 60.9 ± 7.6%. The ratio between the thicknesses of non-compacted and compacted myocardium (NC: C ratio) from long-axis views (LAX) at the end of diastole was 2.54 ± 0.36. The most common non-compaction affected segment was the apical wall of the left ventricle. Left ventricle enlargement was evaluated in four patients (4/8,50%) (Table 2). One patient underwent a CMR examination which revealed a left ventricular excessive trabeculation compatible with hypertrabeculation and the NC/C ratio was 3.4/1 in the end-diastolic. Seventeen segments of the myocardium were identified using the American Heart Association (AHA) standard model (Fig. 2H).

**Table 2:** Baseline clinical, echocardiographic and genetic characteristics of eight LQTS-LVHT co-phenotype patients

Case No.	Case 1	Case 2	Case 3	Case 4	Case 5	Case 6	Case 7	Case 8
Family No.	Family 1	Family 2	Family 2	Family 3	Family 4	Family 5	Family 6	Family 6
General/symptoms								
Age of onset, year	12	8	27	15	6	9	1	1
Age at diagnosis, year	14	21	27	15	10	9	12	5
Sex	Female	Female	Female	Male	Female	Male	Female	Female
Symptoms	Syncope	Syncope	Syncope	Syncope	Syncope//SCA	Dizziness	Asymptomatic	Asymptomatic
Inducement(s) of Symptoms	Exercise and emotion stress	Climbing stairs and depressed mood	Depressed mood or without inducement	Playing computer games	Emotion stress	-	-	-
Duration of unconsciousness	1–2 min	1 min, aggravating to 5–10 min	4–5 min	10s	10 min	-	-	-
Symptoms frequency	Once/year, increasing to twice/year	Once/month, increasing to four times/month, especially menstrual period	Once/year, decreasing to once/3–4 years	Once/year	Once/year	-	-	-
SCD history	No	Yes	Yes	No	No	No	No	No
Echocardiography								
LVEF( %)	53	72	65	52	69	63	59	54
LVEDd(mm)	53↑	49	49	61↑	47	46	57↑	42↑
Non-ncompaction affected segments	apical wall of left ventricular	apical, lateral, inferior and posterior wall of left ventricular	apical, lateral and posterior wall of left ventricular	apical wall of left ventricular	inferior posterior and lateral wall of left ventricular	posterior and lateral wall of left ventricular	inferior posterior of left ventricular	inferior wall of left ventricular
NC/C ratio	2.4/1	2.43/1	2.1/1	2.7/1	2.75/1	3.25/1	2.17/1	2.54/1
Fulfilled CMR criteria for LVHT	-	Yes	-	-	-	-	-	-
Genetic								
Genotype	LQT-7	LQT-2	LQT-2	LQT-2 LQT-3	JLNS	LQT-1	LQT-1	LQT-1
Genetic test	KCNJ2 p.V93I	KCNH2 p.T273M	KCNH2 p.T273M	KCNH2 p.G626S; SCN5A p.A1428S	KCNQ1 p.R174C	KCNQ1 c.1032 + 6T > C	KCNQ1 p.Arg513His	KCNQ1 p.Arg513His
Homozygous/heterozygosis mutations	Heterozygous mutation	Heterozygous mutation	Heterozygous mutation	Heterozygous mutations	Homozygous mutation	Heterozygous mutation	Heterozygous mutation	Heterozygous mutation
Treatment								
Medication/Day	Propranolol 60 mg + mexiletine 450 mg	Propranolol 15 mg + mexiletine 450 mg	Mexiletine 300 mg	Metoprolol 25 mg	Propranolol 15 mg	Propranolol 20 mg	Metoprolol 16 mg	Metoprolol 3 mg
Device implantation	single -chamber ICD	single -chamber ICD	single -chamber ICD	Refused ICD	Refused ICD	No indication	No indication	No indication
Follow-up								
Follow-up Duration (months)	27	32	32	50	29	72	36	36
Cardiac events during follow-up	ICD shock therapy	ICD shock therapy	ICD shock therapy	SCD	SCD	No	Cardiac Syncope	No

Abbreviations: LVEF=left ventricular ejection fraction;LVEDD=left ventricular end-diastolic dimension;CMR=cardiac magnetic resonance;NC/C ratio=ratio of non-compacted and compacted myocardium thicknesses;SCD = sudden cardiac death

### Pedigree characteristics

Amongst 170 genetically-confirmed LQTS patients, eight LQTS-LVHT patients were identified including seven autosomal dominant LQTS [three LQT-1, two LQT-2, one LQT-7, one LQTM (2 and 3)], and one with autosomal recessive LQTS (Jervell and Lange-Nielsen syndrome, JLNS). Moreover, they were from six families (case 2 and 3 were daughter and mother, case 7 and 8 were sisters). No LVHT gene mutations were detected and the genetic test results are shown in Table 2.

**Fig. 3:**
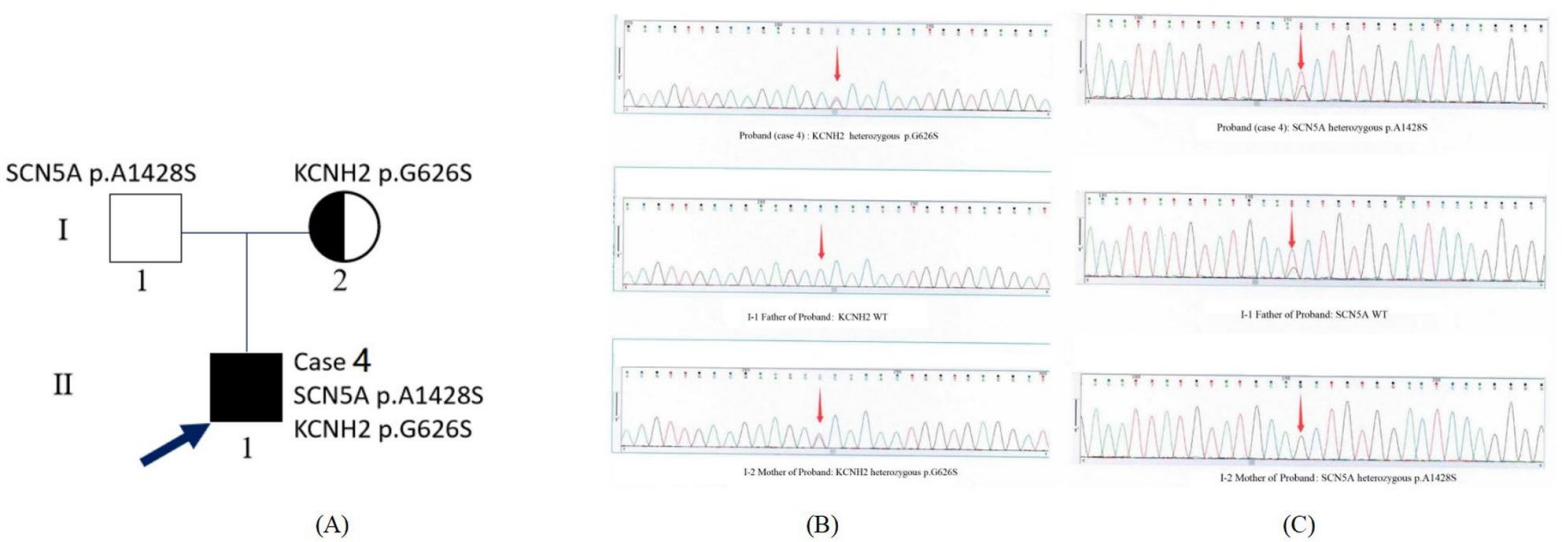
Pedigrees of Case 4(LQTS-LVHT) . The proband(case 4) carried the compound mutation *KCNH2* p.G626S from his mother(I-2) and *SCN5A* p.A1428S from father (I-1). Electrocardiograph showed QTc interval prolonged to 500ms in his mother without any clinical symptoms or cardiac imaging abnormity. The proband’s father was asymptomatic with normal electrocardiograph and echocardiography examination

**Fig. 4:**
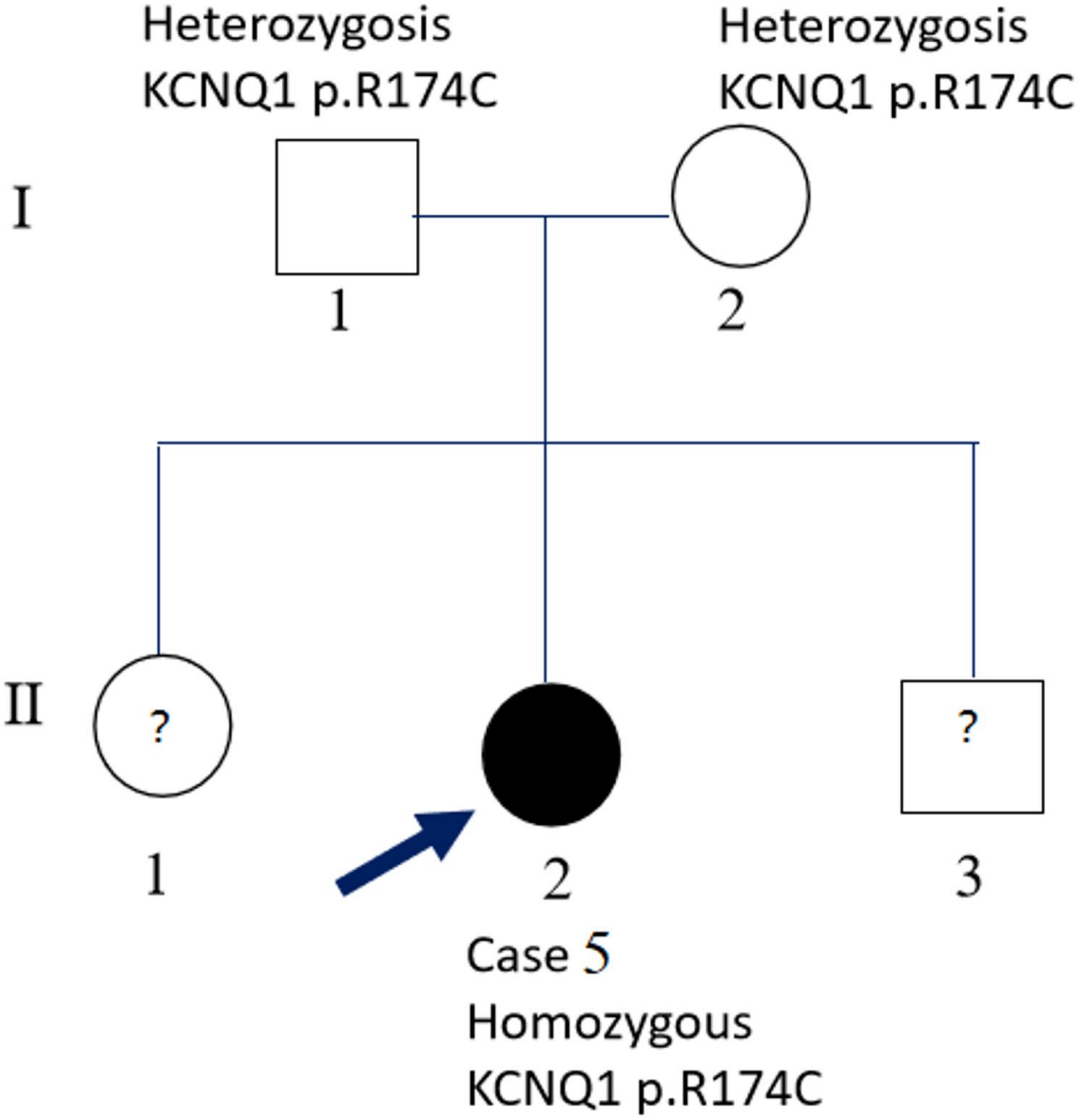
Pedigrees of Case 5 (LQTS-LVHT). Homozygous mutation *KCNQ1* p.R174C was detected in the proband(case 5) with postnatal deaf, and the QTc interval was 716ms with T wave alternans. Heterozygosis mutation *KCNQ1* p.R174C was identified in her parents with no clinical manifestation and normal electrocardiograph

Three family trees are shown in Figs. 2, 3 and 4:

Family of Case 2 and 3 (Fig. 2A-B): The proband (case 2) and her mother (case 3, II-3) were diagnosed with LQTS-LVHT, while the younger sister of the proband (Ⅲ-3) who carries the same mutation *KCNH2* p.T273M was diagnosed with LQTS only (QTc = 614ms).

Family of Case 4 (Fig. 3): The proband (case 4) carried the compound mutation*KCNH2* p.G626S from his mother (1–2) and *SCN5A* p.A1428S from his father (I-1). Electrocardiograph showed QTc interval prolonged to 500ms in his mother without clinical symptom or cardiac imaging abnormality. The proband’s father was asymptomatic with normal electrocardiograph and echocardiography examination.

Family of Case 5 (Fig. 4): Homozygous mutation *KCNQ1* p.R174C was detected in the proband (case 5) with postnatal deaf, and the QTc interval was 716ms with T wave alternans. Heterozygous mutation *KCNQ1* p.R174C was identified in her non-consanguineous parents without clinical manifestation or abnormal electrocardiograph.

### Treatments and follow-up characteristics

In terms of pharmacotherapy, 133 patients (133/170, 78.2%) were prescribed with β-blockers(*n* = 109) and/or mexiletine/flecainide (*n* = 24) in our LQTS cohort. Patients in the LQTS-N-LVHT group who were not on pharmacotherapy were either asymptomatic or at low-risk profiles based on initial evaluation. After the diagnosis, all LQTS-LVHT patients administered β-blockers (*n* = 7) and/or mexiletine (*n* = 3). Three LQTS-LVHT patients received ICD implantation and two patients refused to implant ICD despite strong indications.

**Fig. 5:**
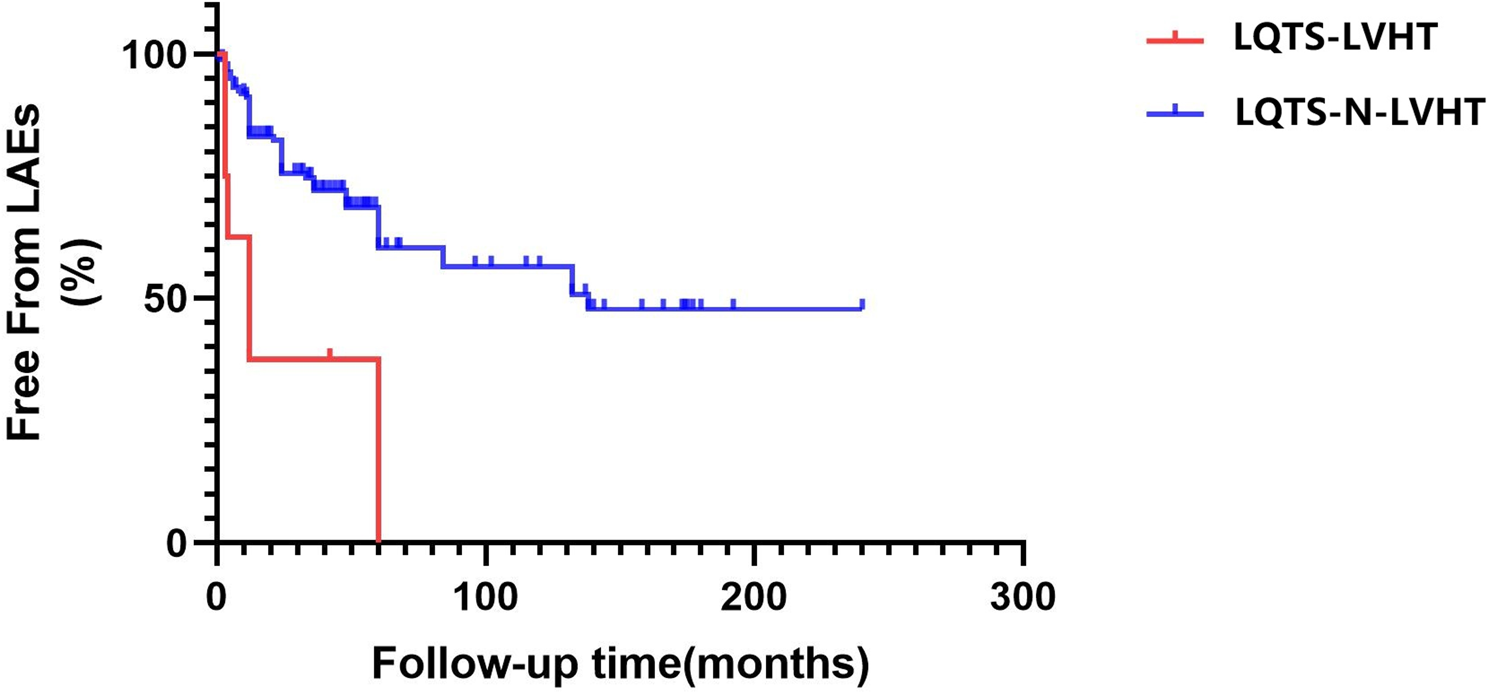
Kaplan-Meier curves demonstrate freedom from LAEs stratified by LVHT. The red line shows percentage of free from LAEs in LQTS-LVHT co-phenotype patients.The blue line indicates percentage of free from LAEs in LQTS-N-LVHT individuals

In total, 59 patients (59/170, 34.7%) developed life-threatening arrhythmic events(LAEs) during follow-up of 52.8 ± 32.3 months. Kaplan-Meier curves demonstrating freedom from LAEs stratified by LVHT are shown in Fig. 5. Significant difference was found between two groups by the log-rank test (*P* < 0.001). A univariate Cox regression analysis was performed demonstrating that LVHT was a significant predictor of LAEs [HR: 4.02 (1.71–9.45), *P* = 0.001].A multivariate Cox regression analysis indicated that LVHT remained a predictor of LAEs [hazard ratio: 3.02 (1.24–7.36), *p* = 0.015] controlled for gender, age of onset, first symptom of cardiac syncope or a resuscitated cardiac arrest, Schwartz score, QTc interval in 12-lead ECGs,β-blocker and mexiletine/flecainide treatment.

## Discussion

Left ventricular hypertrabeculation (LVHT), formerly known as “non-compaction”, is neither definitively classified as a distinct cardiomyopathy nor universally accepted as a benign anatomical variant, consistent with the latest ESC guidelines for cardiomyopathies in 2023 [14]. The etiology of LVHT has not been fully understood, but its genetic inheritance has emerged in at least 30%-50% of patients [2]. LQTS has been considered a very rare lethal disease associated with syncope and sudden death in structurally normal heart. Since 2007, sporadic cases have revealed that LVHT can be co-phenotype with genetic confirmed LQT-1(*KCNQ1* p.D611T, p.L273F) [3, 4] or LQT-2(*KCNH2* p.A561V, p.D501N, p.T1019Pfs×38, p.Y616C) [5–7], or LQTS with *SCN5A* variants [8]. The clinical and genetic characteristics of the reported patients are listed in Table 3.

**Table 3:** The clinical and genetic characteristics of the reported genetic confirmed LQTS probands co-phenotype with LVHT

References	Phenotype	Gene analysis			Age(year)	Symptoms	Heart Rate	QTc(ms)	Ventricular arrhythmia	Other arrhythmia	Medication	Device	Follow up
		Mutation genes	Nucleotide change	Protein change									
Nakashima K 2013 [3]	LQT-1 LVHT	KCNQ1	c.1831 G > T	p. D611T	5	cardiac arrest	76	600	Tdp	-	propranolol and verapamil	-	-
Kharbanda M 2017 [4]	LQT-1 LVHT	KCNQ1	c.817 C > T	p.L273F	48	No	43	512	-	-	-	-	-
Ogawa K 2008 [5]	LQT-2 LVHT	KCNH2		p.A561V	1-day	tachypnea and systemic cyanosis.	103	590	Tdp	-	BB	-	-
	LQT-2 LVHT	KCNH2		p.D501N	5	Syncope	50	560	Tdp	-	BB	-	-
Alsenaidi K S 2014 [6]	LQT-2 LVHT	KCNH2	Homozygous c.3504delC	p.T1019Pfs × 38,	5	Syncope	63	612	Tdp	intermittent 2:1 AV block, IRBBB	BB	-	No VT
Hairui Sun 2022 [7]	LQT-2 LVHT	KCNH2	Heterozygous c.1847 A > G	p.Tyr616Cys	fetus	-	113	-	-	2:1 AV block	-	-	terminate pregnancy
Lishen Shan 2007 [8]	LQT LVHT	SCN5A	87G > A 5457T > C		13	-	-		-	-	-	-	-

Abbreviations: CAVB, complete atrioventricular block; Age, age at clinical evaluation; BB, b-blocker; IRBBB, incomplete right bundle branch block;ICD,implantable cardioverter-defibrillator; PM, pacemaker; SSS, sick sinus syndrome; AVNRT, atrioventricular nodal reentrant tachycardia;PVCs, premature ventricular contraction; bVT, bidirectional ventricular tachycardia; pVT, polymorphic ventricular tachyarrhythmias; Tdp, torsade de points

In our cohort, recurrent syncope was the most popular symptom in eight patients diagnosed with LQTS-LVHT. Patients were easily misdiagnosed with epilepsy because of the seizure-like convulsions. The frequency of syncope increased significantly after menophania and decreased after menopause in female patients, perhaps because estrogen levels affect the function of ion channels (such as inhibiting the Ikr channel) [15]. In the LQTS-LVHT patients, QTc interval prolongation and T wave alternations increased transmural dispersion of repolarization. Additionally, microvascular dysfunction and subendocardial fibrosis could promote re-entry circuits, and Purkinje fiber conduction was altered due to structural disarray [16].These factors may collectively result in the high risk of ventricular arrhythmia and sudden cardiac events. 24-h ambulatory Holter monitoring showed a high-value of capturing Tdp/VF.

In our study, eight LQTS-LVHT patients were from six families. The genetic test of case 1(LQT-7) revealed *KCNJ2* p.V93I. The mutation was firstly reported in Chinese familial atrial fibrillation patients with normal QT interval [17]. It is the first report of a new phenotype with QTc prolongation and Tdp induced by a burst stimulation during electrophysiological examinations. The same genotype *KCNH2* p.T273M was identified in case 2,3 and patient III-3 (family 1), with a different phenotype of LVHT. The proband with LVHT had more serious clinical symptoms and malignant ventricular arrhythmia after optimized medication (propranolol, mexiletine) treatment compared with her sister who does not have LVHT. Case 4 carried a combination of mutation *KCNH2* p.G626S from his mother and *SCN5A* p.A1428S from his father. Schwartz et al. [18] reported that 6/130 (4.6%) LQTS probands had the compound or digenic mutations, and probands who have a digenic or compound mutation experience QTc prolongation with severe symptoms like syncope and cardiac collapse. *KCNH2* p.G626S was described to have loss-of-function effects on the pore region of the IKr channel and acts as a disease-causing variant [19]. *SCN5A* A1428S was reported in a LQT3 patient and a repeated-syncope child with a combination of compound mutations (*KCNH2* N45D, *SCN5A* A1428S) and gene variant in *KCNE1*(D85N) [20]. Furthermore, LVHT worsened clinical symptoms in the occurrence of sudden cardiac death. Jervell and Lange-Nielsen syndrome (JLNS), an autosomal-recessive variant of long QT syndrome characterized by deafness, significant QT prolongation (usually more than 500 ms), and a high risk of sudden death, is brought on by homozygous or compound heterozygous mutations in *KCNQ1*. The homozygous *KCNQ1* c.520 C > T (p.R174C) mutation of case 5 was detected. One non-consanguineous Turkish family with JLNS previously reported compound heterozygosity for missense (c.520 C > T, p.R174C) and splicing site (c.477 + 1G > A) in the *KCNQ1* gene [21]. This missense mutation *KCNQ1* R174C is positioned in the S2 domain of the protein and strikes the S2/S3 domains, disrupting the gating function of the ion channel. Electrophysiological study of *KCNQ1* R174C alone produced no IKs currents, however the steady-state and tail IKs amplitude under WT (wild-type) + R174C circumstances were slightly reduced compared to WT alone [22]. That is probably the cause of the heterozygous carriers’ lack of symptoms and significant extended QTc interval.

Four stages make up the formation of the ventricular wall. At an early developmental stage known as embryonic day (E) 8.5, a single cell layer of myocardium is formed. At an early midgestational stage known as E9.5-10.5, a trabeculated and compact myocardium is formed. During an advanced midgestational stage known as E12.5-13.5, myocardial compaction occurs. At an advanced fetal and neonatal stage known as E14.5-18.5, a mature and multilayered spiral myocardium. In mouse embryos, the majority of trabecular have compacted by E14.5 [23].

Cardiac ion channel genes are expressed in the mouse heart during heart development. At E9.5, the mRNAs (and protein) for *KCNQ1* and *KCNH2* are initially expressed [24]. At stage E11.5, the *SCN5A* mRNA levels reached a peak. They dropped during the following stages and then gradually increased starting at E17.5 [25]. *SCN5A* mRNA displayed an increased expression during the third stage in the conducting system and trabeculated myocardium. Kir2.1 (*KCNJ2* encodes) plays a vital regulation role in the cardiac inward-rectifier K^+^ currents during the prenatal development stage E14 [26].

Ventricular hypertrabeculation is hypothesized to be related to abnormal trabecular remodeling (i.e. compaction) during ventricular wall development. Therefore, *KCNQ1*, *KCNH2*, *SCN5A*, and *KCNJ2* channels are expressed at the same time as the ventricular trabecular occurs or remodels, so changes to the channels’ structure and function could harm ventricular trabecular development during the embryonic stage.

We identified family members of the probands having the same *KCNH2* mutations, despite the fact that none of the probands’ relatives showed any echocardiographic signs of LVHT. The potential role of acquired variables and the relationship between genotype and phenotype need to be further examined by future studies.

### Prognosis

It can be concluded that the risk of life-threatening arrhythmic events is high when LQTS and LVHT coexist in patients. Also, there is a strong indication of ICD implantation for recurrent syncope patients with malignant ventricular arrhythmia despite optimized medication therapy. However, limited by low economic status and poor acceptance of implanting devices in China, two patients in our cohort experienced SCD events after refusing ICD implantation. Additionally, the sympathetic denervation in the LVHT-LQTS patients might be a promising adjunctive therapy for arrhythmia prevention. Consequently, more focus should be placed on the evaluation of cardiac structures by echocardiography and/or CMR. When LVHT is detected, it is necessary to educate the patients on regular medication and device treatment to prevent SCD.

### Limitations

It is generally unknown how these variations affect the co-phenotype LVHT-LQTS patients and what mechanisms underlie those effects. Further studies of ion channel functions and cardiac development may offer new insights.

## Conclusion

The rate of the life-threatening arrhythmic event was higher for LQTS patients who have LVHT dignosis.LVHT is a novel predictor of life-threatening arrhythmic events in LQTS patients. Effective prevention and management should be emphasized to prevent life-threatening cardiac events in LQTS-LVHT patients.

## Supplementary Information

Below is the link to the electronic supplementary material.

Supplementary Material 1

## Data Availability

The article/Supplementary material contains the original data used for this investigation; further questions should be addressed to the corresponding author

## Abbreviations

LQTS: Long QT syndrome
LVHT: Left ventricular hypertrabeculation
LAEs: Life-threatening arrhythmic events
CMR: Cardiac magnetic resonance imaging
EF: Ejection fraction
ICD: Implantable cardioverter defibrillator
SCD: Sudden cardiac death
