# Supplementary material for: Left ventricular hypertrabeculation is a novel predictor of life-threatening arrhythmic events in long QT syndrome patients
Source: Orphanet J Rare Dis. 2025 Dec 22;20:634. doi: 10.1186/s13023-025-04172-7 (PMC12751391; doi:10.1186/s13023-025-04172-7)
Supplement: Supplementary file 1 — Supplementary Material 1 [file 13023_2025_4172_MOESM1_ESM.docx]

Supplementary Material

## Supplementary Tables

**Supplementary Table 1: References of LVHT diagnostic criteria**

| References | Modality | LVHT diagnostic criteria |
| --- | --- | --- |
| Jenni et al1**^[10]^** | Echo | 1. 2-layered structure with a compacted epicardial and noncompacted endocardial layer 2. Color Doppler evidence of intertrabecular recesses supplied by intraventricular blood, absence of coexisting cardiac structural abnormalities 3. End-systole NC/C layer≥2 |
| Petersen SE et al**^[11]^** | CMR | 1. 2-layered structure with a compacted epicardial and noncompacted endocardial layer 2. Images from horizontal and long-axis views at points of prominent trabeculations 3. End-diastolic NC/C>2.3 |

Echo=Echocardiogram; CMR= cardiac magnetic resonance; NC/C=maximum noncompaction to compaction ratio;
